# Respiratory mechanics and patient–ventilator interaction dataset from the ICU

**DOI:** 10.1038/s41597-026-07598-1

**Published:** 2026-06-10

**Authors:** Verónica Santos-Pulpón, Francesc Suñol, Josefina López-Aguilar, Candelaria de Haro, Sol Fernández-Gonzalo, Gemma Gomà, Emilio Diaz Santos, Robert Huhle, Jakob Wittenstein, Lluís Blanch, Leonardo Sarlabous

**Affiliations:** 1 Critical Care Department, Parc Taulí Hospital Universitari, Institut d’Investigació I Innovació Parc Taulí (I3PT-CERCA), Universitat Autònoma de Barcelona, Sabadell, Spain; 2 Physics Department, Universitat Politècnica de Catalunya - BarcelonaTech, 08034 Barcelona, Spain; 3 Centro Investigación Biomédica en Red de Enfermedades Respiratorias (CIBERES), Instituto de Salud Carlos III, Madrid, Spain; 4 Centro Investigación Biomédica en Red de Salud Mental (CIBERSAM), Instituto de Salud Carlos III, Madrid, Spain; 5 Department of Anesthesiology and Intensive Care Medicine, Faculty of Medicine and University Hospital Carl Gustav Carus, TUD Dresden University of Technology, Dresden, Germany

## Abstract

The use of high-resolution data in the intensive care unit (ICU) environment allows for more detailed research into invasive mechanical ventilation (IMV) management and patient–ventilator interactions and their relationship with clinical outcome. The Real-time Agnostic Monitoring for Intensive Care (RAMIC–I) database is a single-center, de-identified dataset containing clinical and physiological data collected from 155 critically ill adults admitted to the ICU of Hospital Universitari Parc Taulí (Sabadell, Spain), between 2015 and 2019. RAMIC–I includes 13,433 hours of mechanical ventilation recorded during the first eight days of IMV, enriched with demographic information, bedside clinical variables (including clinical management variables, administered treatments, and clinical scores), vital signs, and mortality outcomes. Derived variables from respiratory waveforms, including patient-ventilator asynchronies, were aggregated into 10-minute resolution intervals to enable a comprehensive exploration of respiratory physiology during IMV. The dataset is structured according to FAIR data principles and is accessible through a federated repository based on the open-source infrastructure. This detailed resource supports research into predictive modeling, IMV optimization, and the study of patient–ventilator interactions in critical care settings.

## Background & Summary

The collection and organization of clinical data in the Intensive Care Units (ICUs) have evolved from manual, paper-based records to electronic systems capable of capturing numerous parameters in real time. This digital transformation has introduced challenges related to data integration, standardization, and management of large, heterogeneous data streams^1,2^. Database initiatives such as MIMIC-III/IV, the eICU Collaborative Research Database, AmsterdamUMCdb, HiRID, and SICdb have provided crucial resources for advancing critical care research^1,3–6^. These resources have supported a wide range of studies by providing structured, de-identified data from diverse ICU settings^7^.

Despite the significant contributions of these databases, limitations remain in the granularity of ventilator waveform data and the standardized collection of detailed mechanical ventilation parameters. These limitations restrict research opportunities for investigating patient-ventilator interactions and respiratory mechanics in critically ill patients receiving invasive mechanical ventilation (IMV)^8–10^.

To facilitate research in this area, we describe here a structured, de-identified dataset comprising detailed respiratory mechanics and clinical parameters, collected over up to eight days of IMV from adult patients admitted to the ICU at Parc Taulí Hospital Universitari. The dataset includes respiratory and patient–ventilator interaction metrics derived from high-frequency respiratory waveforms, alongside demographic information, vital signs, and clinical outcomes. The database is intended to augment existing critical care resources by enabling research focused on the analysis of patient–ventilator interaction during IMV^11,12^.

By providing access to high-resolution clinical and physiological data, this dataset may contribute to multidisciplinary efforts in intensive care medicine, including applications in predictive modeling and the study of precision and patient-tailored respiratory support.

## Methods

### Study design and patient selection

The Real-time Agnostic Monitoring for Intensive Care (RAMIC–I)^13^ includes de-identified clinical data from 155 adult patients admitted to the ICU at Hospital Universitari Parc Taulí (Sabadell, Spain) between 2015 and 2019. All patients required IMV for at least 24 consecutive hours. The study protocol was approved by the I3PT institutional Research Ethics Committee (Code: 20225098 and ClinicalTrials.gov ID: NCT03451461, NCT02714751). Individual informed consent was waived as data collection did not interfere with clinical care and patient data were anonymized prior to inclusion.

Inclusion criteria included admission to ICU beds equipped with the BCLink system platform, age ≥18 years, and duration of IMV ≥24 hours. Patients were included only if IMV waveforms data were available for the analysis period. ICU bed assignment was conducted by clinical staff as part of routine care, based on bed availability and patient needs, and was independent of this study. Only beds equipped with the BCLink system were eligible; therefore, inclusion in the dataset depended on admission to these instrumented beds rather than on study-driven selection. This approach avoided intentional selection by the research team, although it does not preclude potential selection bias.

Exclusion criteria included the presence of chest tubes with suspected bronchopleural fistula, absence of mechanical ventilation waveforms during the observation period, technical issues affecting signal synchronization or integrity, and absence of physiological signals within the predefined target window.

### Data acquisition

Physiological waveforms, including mechanical ventilation tracings and vital signs, were continuously recorded at 200 Hz using the BCLink System (BetterCare™, Barcelona, Spain; U.S. Patent No. 12/538,940), which interfaces with mechanical ventilators and bedside monitors to capture synchronized signals. RAMIC–I^13^ includes 13,433 hours of mechanical ventilation waveform data, corresponding to 53.7% of the maximum achievable recording time across the cohort during the first 8-day following IMV initiation, representing approximately 19 665 086 breaths. This maximum achievable duration was calculated from the actual IMV exposure of each patient (capped at 8-day), yielding approximately 25,032 hours, compared with an absolute theoretical upper bound of 29,760 hours if all 155 patients had remained ventilated for the full 8-day window. The remaining difference between the maximum achievable and recorded duration reflects both inter-patient variability in IMV duration and periods of missing or unavailable waveform data related to technical or acquisition factors. Consequently, respiratory waveform availability during the first 8-day after IMV initiation was heterogeneous across patients and study days.

**Fig. 1:**
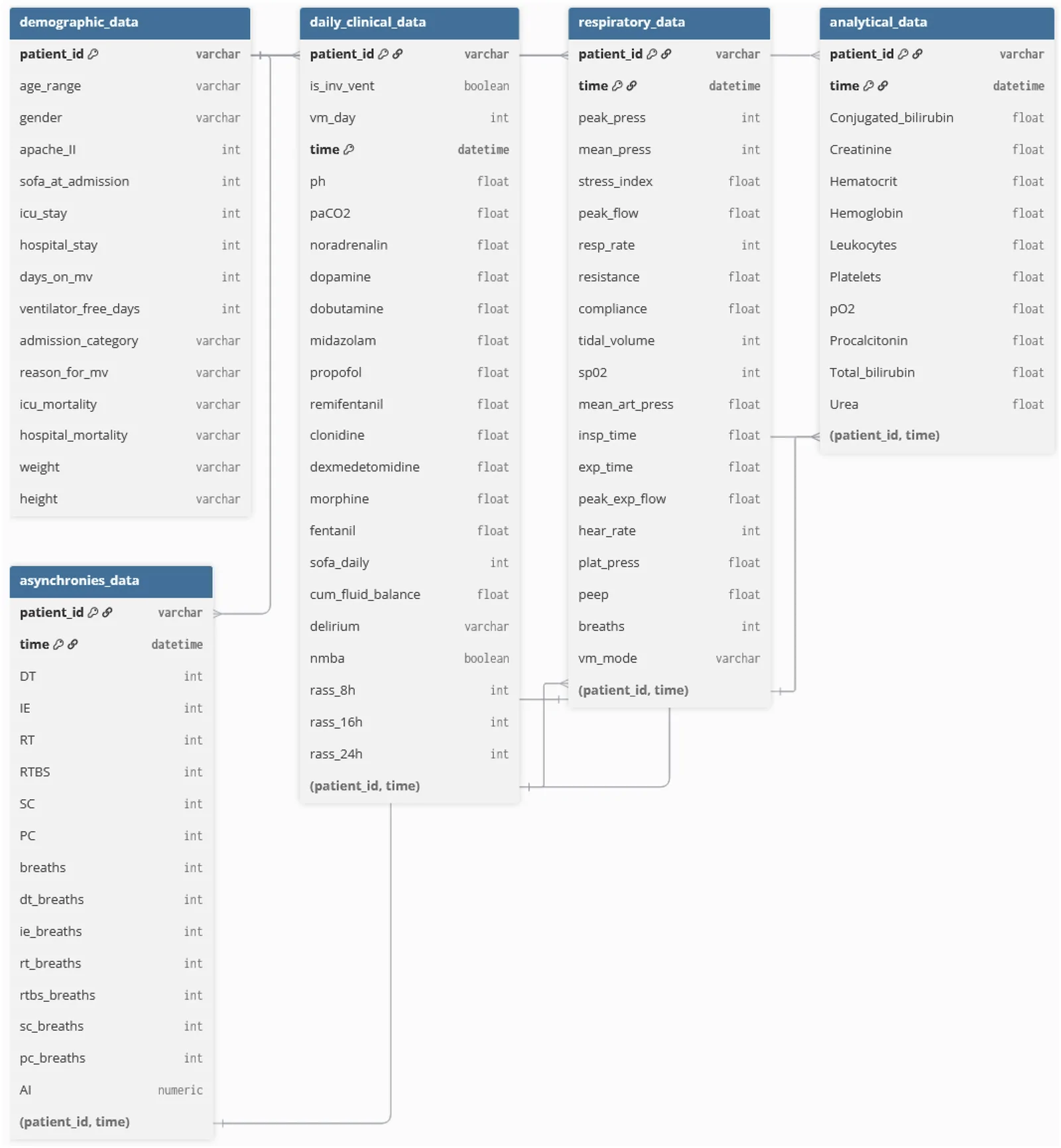
RAMIC- I database diagram.

Signal processing algorithms were applied to the physiological waveforms to extract breath-by-breath respiratory parameters and patient–ventilator asynchrony events (see Fig. 1), which were stored in a PostgreSQL (pgAdmin 4, version 7.8) relational database. Subsequently, these parameters were aggregated into non-overlapping 10-minute intervals, with one representative value per variable stored for each interval.

Figure 1 illustrates the structure of data tables containing demographic, clinical and analytical variables extracted from the hospital electronic medical record system. These variables were recorded with different temporal resolutions, enabling the analysis of both short-term fluctuations in respiratory parameters and longer-term trends in clinical and analytical data. Demographic variables were recorded one per patient for the entire ICU stay. Clinical variables (including clinical management variables, administered treatments, and clinical scores) were documented once daily. Laboratory variables (measurements obtained from biological sample analyses) included multiple daily entries for some parameters, whereas others were limited to a single value per day. Respiratory and asynchronies variables (respiratory_data and asynchronies_data tables) were summarized into non-overlapping 10-minute intervals, with one representative value (mean and count, respectively) per variable stored for each interval.

**Fig. 2:**
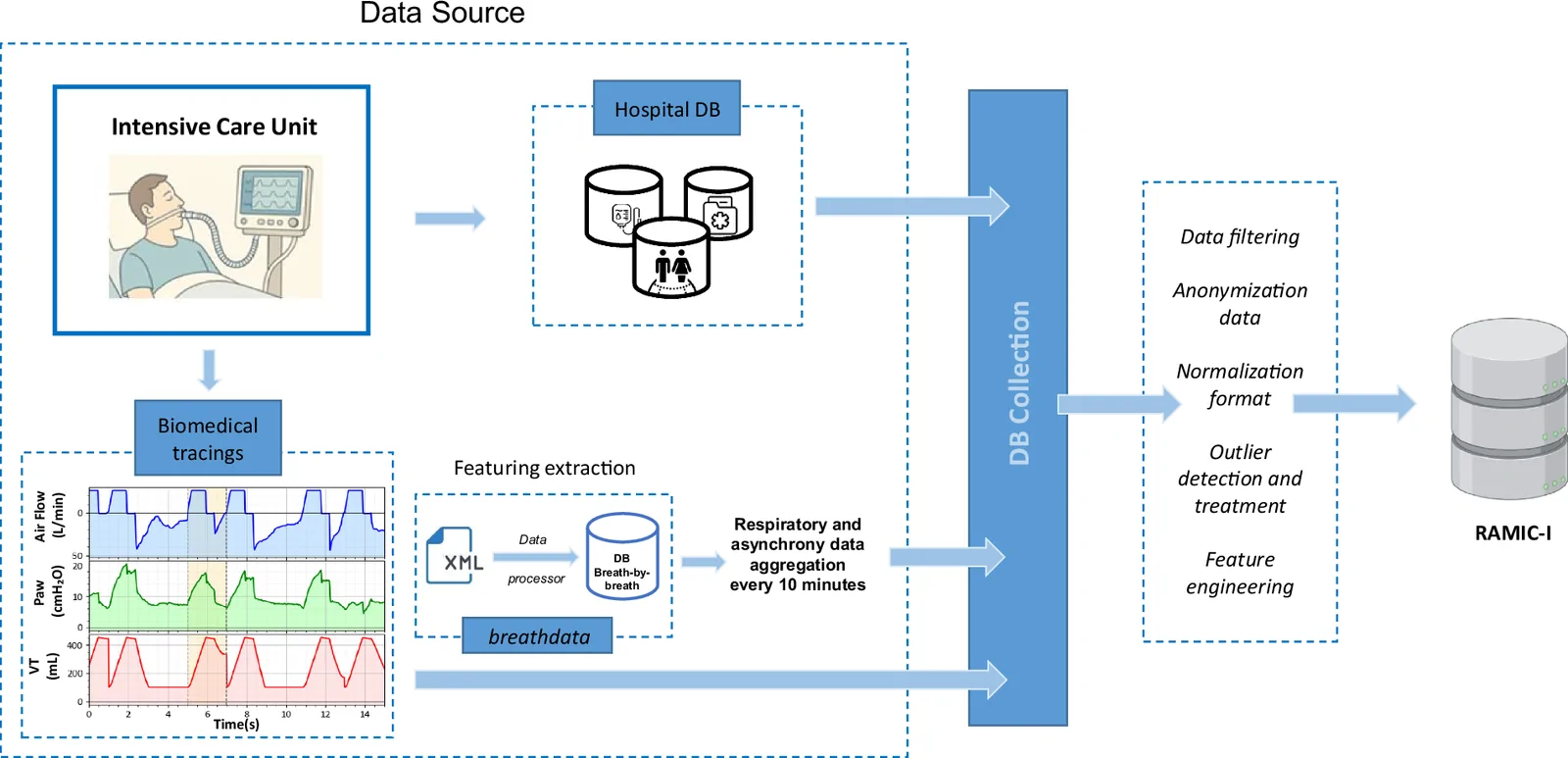
Pathway of data from acquisition to storage. At the ICU bedside, physiological tracings are continuously recorded from intubation to extubation of the patient from IMV. The dataset undergoes successive steps of filtering, anonymization to protect patient identity, normalization to standardize variable formats, outlier detection and correction, and feature engineering to enhance data usability.

Preprocessing steps (Fig. 2) were performed using custom Python scripts (Python v3.9.7) and included harmonization of missing-value representations, timestamp standardization, variable name consistency checks, and physiological plausibility filtering. Respiratory waveform-derived variables were screened using predefined admissible ranges established by experienced intensive care clinicians, and values outside these ranges were replaced with missing values (NaN). No winsorization, percentile clipping, normalization, scaling, or imputation procedures were applied. Original measurement units and clinically interpretable values were preserved to facilitate downstream reuse and task-specific preprocessing. All code was executed on a desktop computer running Windows 10 Pro 64-bit, with an Intel®Core(TM) i7-6700 CPU @ 3.40 GHz and 16GB RAM.

### Data collection timeline

The dataset includes data uniformly collected over an 8-day time window of IMV for each patient, to maintain consistency and comparability in longitudinal analyses. Although the average ICU length of stay was approximately 11 days^14,15^, the 8-day window was selected to focus on the early and most clinically relevant phase of IMV, which typically occurs soon after ICU admission^16,17^. In the RAMIC-I^13^ cohort, 66.5% of patients received less than 11 days of IMV.

Future developments include the planned expansion to RAMIC-II, extending coverage to the entire ICU stay as well as post-ICU functional and neuropsychological outcomes, thereby enabling broader temporal analyses and addressing a wider range of research questions. Moreover, upcoming dataset releases will incorporate comprehensive post-ICU assessments, facilitating longitudinal follow-up of ICU survivors and supporting novel research directions in post-intensive care syndrome (PICS) and long-term recovery outcomes.

### Data processing pipeline

Figure 2 illustrates the data flow architecture of the RAMIC-I^13^ database, describing the process from data acquisition in ICU to the final storage in the relational database. Data sources include continuous physiological waveform recordings obtained from physiological tracings at the bedside, clinical and analytical data extracted from the hospital electronic medical record system (Health Care Information System and Centricity High Acuity critical care), and critical care information system archives. These datasets are combined with patient demographic and clinical variables in a data collection pipeline.

### Patient-ventilator asynchronies description

**Fig. 3:**
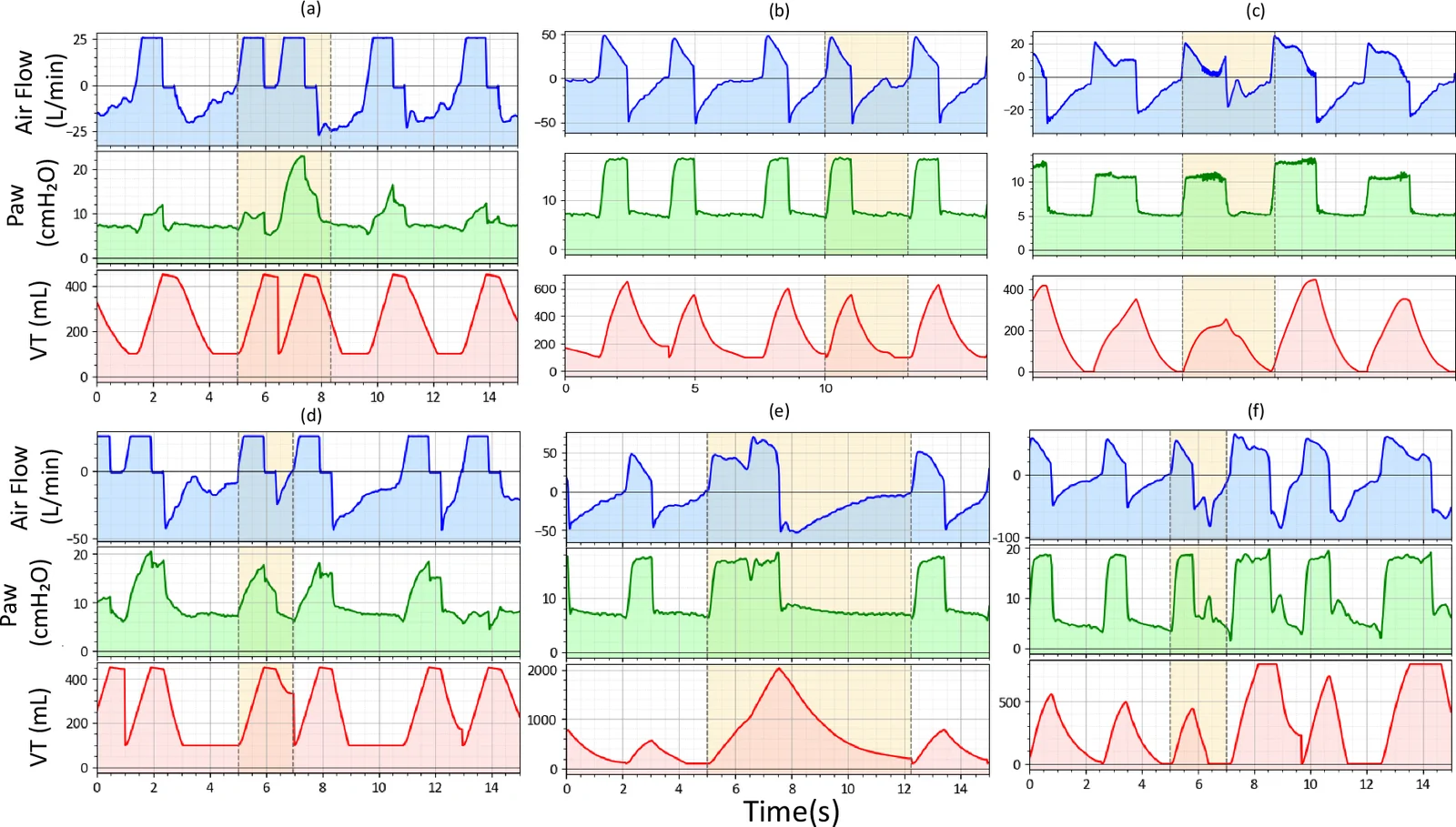
Graphical representation of patient-ventilator asynchronies. (**a**) Double Trigger; (**b**) Ineffective Effort; (**c**) Reverse Triggering; (**d**) Reverse Triggering with Breath Stacking; (**e**) Prolonged Cycling; (**f**) Short Cycling.

The dataset includes counts of patient–ventilator asynchronies comprising double triggering (DT)^18–20^, ineffective effort (IE)^21,22^, reverse triggering (RT)^23^, reverse triggering with breath stacking (RTBS)^24^, short cycling (SC)^25,26^, and prolonged cycling (PC)^25,26^. Definitions and examples of patient-ventilator asynchronies are also available in several studies conducted in human subjects^11,19,24,27^ and in porcine models^28,29^. Figure 3 provides a detailed graphical representation of patient-ventilator asynchronies, illustrating the distinct patterns and characteristics associated with each asynchrony type. Asynchronies were detected using algorithms implemented in the BetterCare™ software.

In addition, the asynchrony index (AI)^23^, which quantifies the proportion of breaths affected by asynchronies within each 10-minute interval, was calculated using the formula:

$${\rm{AI}}( \% )=\frac{({\rm{DT}}+{\rm{IE}}+{\rm{RT}}+{\rm{RTBS}}+{\rm{SC}}+{\rm{PC}})}{{\rm{Total}}\,{\rm{breath}}\,{\rm{count}}+{\rm{IE}}}\times 100$$

### Data management and anonymization

To ensure compliance with privacy regulations and protect patient confidentiality, data underwent thorough anonymization^30^. Direct identifiers defined by HIPAA were removed. Each patient’s admission dates were shifted to a random fictitious period between 2100 and 2200, preserving the seasonal rhythm. Age was grouped into five-year intervals and capped at 90 years to minimize re-identification risk. Measurement units were standardized to prevent inadvertent disclosure of admission timing^31,32^.

## Data Record

### Repository and data access

The RAMIC-I^13^ dataset is hosted in the institutional research repository “Repositorio de Datos de Investigación (RDR)”, a federated repository managed by the Consorcio de Servicios Universitarios de Cataluña (CSUC) and based on the open-source Dataverse platform. The dataset is accessible at https://doi.org/10.34810/data2509.

Data are provided in tabular format (.tab), facilitating straightforward download and local analysis using common statistical or data science tools. Researchers can directly access and download the dataset openly through HTTPS without registration or access barriers.

The RDR platform ensures long-term digital preservation, persistent DOI-based citation, and full metadata interoperability across international repositories through the federated Dataverse network, thereby supporting sustainable, transparent, and FAIR-aligned data sharing (Findable, Accessible, Interoperable, and Reusable).

### Dataset structure and file format

The RAMIC-I^13^ dataset in RDR comprises five main tables available exclusively in the tabular format (.tab) to facilitate accessibility and compatibility with diverse analytical tools.

The dataset integrates variables collected at different temporal resolutions depending on their source and acquisition workflow. Consequently, the interpretation of the timestamp field varies across tables. For the respiratory_data and asynchronies_data tables, each row corresponds to a single non-overlapping 10-minute observation interval per patient. The aggregate values were calculated from the valid breath-by-breath observations available within that interval. If no valid source data were available during a given 10-minute window, no row was generated for that interval. The timestamp indicates the start of that interval, and one aggregated value per variable is provided for each 10-minute window. For the daily_clinical_data table, the timestamp corresponds to the calendar day on which bedside clinical information was documented. For the analytical_data table, the timestamp reflects the time at which the laboratory result was recorded in the hospital information system, resulting in an event-based and irregular temporal resolution. Demographic variables are static patient-level descriptors recorded once per admission and therefore do not follow a repeated time-series structure.

A detailed and standardized description of all variables, including definitions, units of measurement, data provenance, and temporal resolution, is provided in Tables 1–5, and should be interpreted within the acquisition framework described above.

**Table 1:** Demographic and outcome variables from ramic_demographic_data table.

Variable	Description	Unit	Provenance	Temporal Resolution
patient_id	Unique anonymized patient identifier	categorical	Generated through dataset anonymization process	Static
age_range	Age category derived from exact patient age during anonymization	years (range)	Derived from ICU clinical record	Static
gender	Patient sex	categorical	Centricity High Acuity critical care (CHA)	Static
apache_II	APACHE II score at ICU admission	score	CHA/Health Care Information System (HCIS)	At admission
sofa_at_admission	SOFA score at ICU admission	score	CHA/HCIS	At admission
icu_stay	Length of ICU stay	days	CHA	Final outcome
hospital_stay	Total hospital stay duration	days	HCIS	Final outcome
days_on_mv	Total duration of invasive mechanical ventilation	days	CHA	Final outcome
ventilator_free_days	Days alive and free from mechanical ventilation at day 28	days	Derived outcome	Fixed endpoint (day 28)
admission_category	Main ICU admission category	categorical	CHA	At admission
reason_for_mv	Primary reason for initiating IMV	categorical	CHA	At admission
icu_mortality	Death during ICU stay	binary	CHA	Final outcome
hospital_mortality	Death during hospital stay	binary	HCIS	Final outcome
weight	Patient body weight	kg	CHA	At admission
height	Patient height	cm	CHA	At admission

**Table 2:** Daily clinical variables from dailyclinical_data table.

Variable	Description	Unit	Provenance	Temporal Resolution
**patient_id**	Unique anonymized patient identifier	categorical	Generated through dataset anonymization process	Static
**is_inv_vent**	Receiving invasive mechanical ventilation	binary (0/1)	Centricity High Acuity critical care (CHA)	Daily
**vm_day**	Day since IMV initiation	days	CHA	Daily
**time**	Timestamp (calendar day of record)	datetime	CHA	Daily
**ph**	Arterial blood pH	pH units	Health Care Information System (HCIS)/ Point-of-care	Daily
**paco2**	Arterial carbon dioxide partial pressure	mmHg	HCIS/Point-of-care	Daily
**noradrenalin**	Noradrenaline dose	µg/kg/min	CHA	Daily
**dopamine**	Dopamine dose	µg/kg/min	CHA	Daily
**dobutamine**	Dobutamine dose	µg/kg/min	CHA	Daily
**midazolam**	Midazolam dose	mg/h	CHA	Daily
**propofol**	Propofol dose	mg/h	CHA	Daily
**remifentanil**	Remifentanil dose	µg/kg/min	CHA	Daily
**clonidine**	Clonidine dose	µg/h	CHA	Daily
**dexmedetomidine**	Dexmedetomidine dose	µg/kg/h	CHA	Daily
**morphine**	Morphine dose	mg/h	CHA	Daily
**fentanil**	Fentanyl dose	µg/h	CHA	Daily
**sofa_daily**	Daily Sequential Organ Failure Assessment score	score	CHA/HCIS	Daily
**cum_fluid_balance**	Cumulative fluid balance	mL	CHA	Daily
**delirium**	Presence of delirium	binary (0/1)	CHA	Daily
**nmba**	Use of neuromuscular blocking agents	binary (0/1)	CHA	Daily
**rass_8h**	Richmond Agitation–Sedation Scale at 8 h	score (−5 to +4)	CHA	Daily
**rass_16h**	Richmond Agitation–Sedation Scale at 16 h	score (−5 to +4)	CHA	Daily
**rass_24h**	Richmond Agitation–Sedation Scale at 24 h	score (−5 to +4)	CHA	Daily

**Table 3:** Laboratory variables from analytical_data table.

Variable	Description	Unit	Provenance	Temporal Resolution
patient_id	Unique anonymized patient identifier	categorical	Generated through dataset anonymization process	Static
time	Timestamp of laboratory result registration	datetime	Health Care Information System (HCIS)	Event-based
Conjugated_bilirubin	Direct bilirubin	mg/dL	HCIS	Event-based
Creatinine	Kidney function marker	mg/dL	HCIS	Event-based
Hematocrit	Fraction of red blood cells	%	HCIS	Event-based
Hemoglobin	Oxygen-carrying protein concentration	g/dL	HCIS	Event-based
Leukocytes	White blood cell count	×10^9^/L	HCIS	Event-based
Platelets	Platelet count	×10^9^/L	HCIS	Event-based
pO2	Arterial oxygen partial pressure	mmHg	HCIS/Point-of-care	Event-based
Procalcitonin	Biomarker of bacterial infection and sepsis	ng/mL	HCIS	Event-based
Total_bilirubin	Total bilirubin (direct + indirect)	mg/dL	HCIS	Event-based
Urea	Urea concentration	mg/dL	HCIS	Event-based

**Table 4:** Respiratory variables from respiratory_data table.

Variable	Description	Unit	Provenance	Temporal Resolution
patient_id	Unique anonymized patient identifier	categorical	Generated through dataset anonymization process	Static
time	Timestamp (start of 10-minute interval)	datetime	Derived from waveform timestamps	10-min interval
peak_press	Peak airway pressure	cmH₂O	Computed from mechanical ventilator waveforms	10-min interval
mean_press	Mean airway pressure	cmH₂O	Computed from mechanical ventilator waveforms	10-min interval
stress_index	Stress index	dimensionless	Computed from mechanical ventilator waveforms	10-min interval
peak_flow	Peak inspiratory flow	L/min	Computed from mechanical ventilator waveforms	10-min interval
resp_rate	Respiratory rate	breaths/min	Computed from mechanical ventilator waveforms	10-min interval
resistance	Respiratory system resistance	cmH₂O/L/s	Computed from mechanical ventilator waveforms	10-min interval
compliance	Respiratory system compliance	mL/cmH₂O	Computed from mechanical ventilator waveforms	10-min interval
tidal_volume	Expired tidal volume per breath	mL	Computed from mechanical ventilator waveforms	10-min interval
SpO₂	Peripheral oxygen saturation	%	Computed from bedside monitor waveforms	10-min interval
mean_art_press	Mean arterial pressure	mmHg	Computed from bedside monitor waveforms	10-min interval
insp_time	Inspiratory time	s	Computed from mechanical ventilator waveforms	10-min interval
exp_time	Expiratory time	s	Computed from mechanical ventilator waveforms	10-min interval
peak_exp_flow	Peak expiratory flow	L/min	Computed from mechanical ventilator waveforms	10-min interval
heart_rate	Heart rate	bpm	Computed from bedside monitor waveforms	10-min interval
plat_press	Plateau airway pressure (estimated)	cmH₂O	Computed from mechanical ventilator waveforms	10-min interval
peep	Positive end-expiratory pressure	cmH₂O	Computed from mechanical ventilator waveforms	10-min interval
breaths	Number of breaths in interval	count	Computed from mechanical ventilator waveforms	10-min interval
vm_mode	Predominant ventilatory mode (>70% of breaths)	Categorical	Computed from mechanical ventilator waveforms	10-min interval

Variables were derived from mechanical ventilator and bedside monitor waveforms aggregated into non-overlapping 10-minute intervals.

**Table 5:** Patient–ventilator asynchrony variables from asynchronies_data table.

Variable	Description	Unit	Provenance	Temporal Resolution
patient_id	Unique anonymized patient identifier	categorical	Generated through dataset anonymization process	Static
time	Timestamp (start of 10-minute interval)	datetime	Derived from waveform timestamps	10-min interval
DT	Double triggering events	count	Computed from mechanical ventilator waveforms	10-min interval
IE	Ineffective effort events	count	Computed from mechanical ventilator waveforms	10-min interval
RT	Reverse triggering events	count	Computed from mechanical ventilator waveforms	10-min interval
RTBS	Reverse triggering breath stacking	count	Computed from mechanical ventilator waveforms	10-min interval
SC	Short cycling events	count	Computed from mechanical ventilator waveforms	10-min interval
PC	Prolonged cycling events	count	Computed from mechanical ventilator waveforms	10-min interval
breaths	Number of breaths in interval	count	Computed from mechanical ventilator waveforms	10-min interval
dt_breath	Proportion of breaths with double triggering	ratio (%)	Derived from event counts	10-min interval
ie_breath	Proportion of breaths with ineffective effort	ratio (%)	Derived from event counts	10-min interval
rt_breath	Proportion of breaths with reverse triggering	ratio (%)	Derived from event counts	10-min interval
rtbs_breath	Proportion of breaths with reverse triggering breath stacking	ratio (%)	Derived from event counts	10-min interval
sc_breath	Proportion of breaths with short cycling	ratio (%)	Derived from event counts	10-min interval
pc_breath	Proportion of breaths with prolonged cycling	ratio (%)	Derived from event counts	10-min interval
AI	Asynchrony index	ratio %	Derived from event counts and total breaths	10-min interval

Variables were automatically computed from mechanical ventilator waveforms using computational algorithms.

## Data Overview

**Fig. 4:**
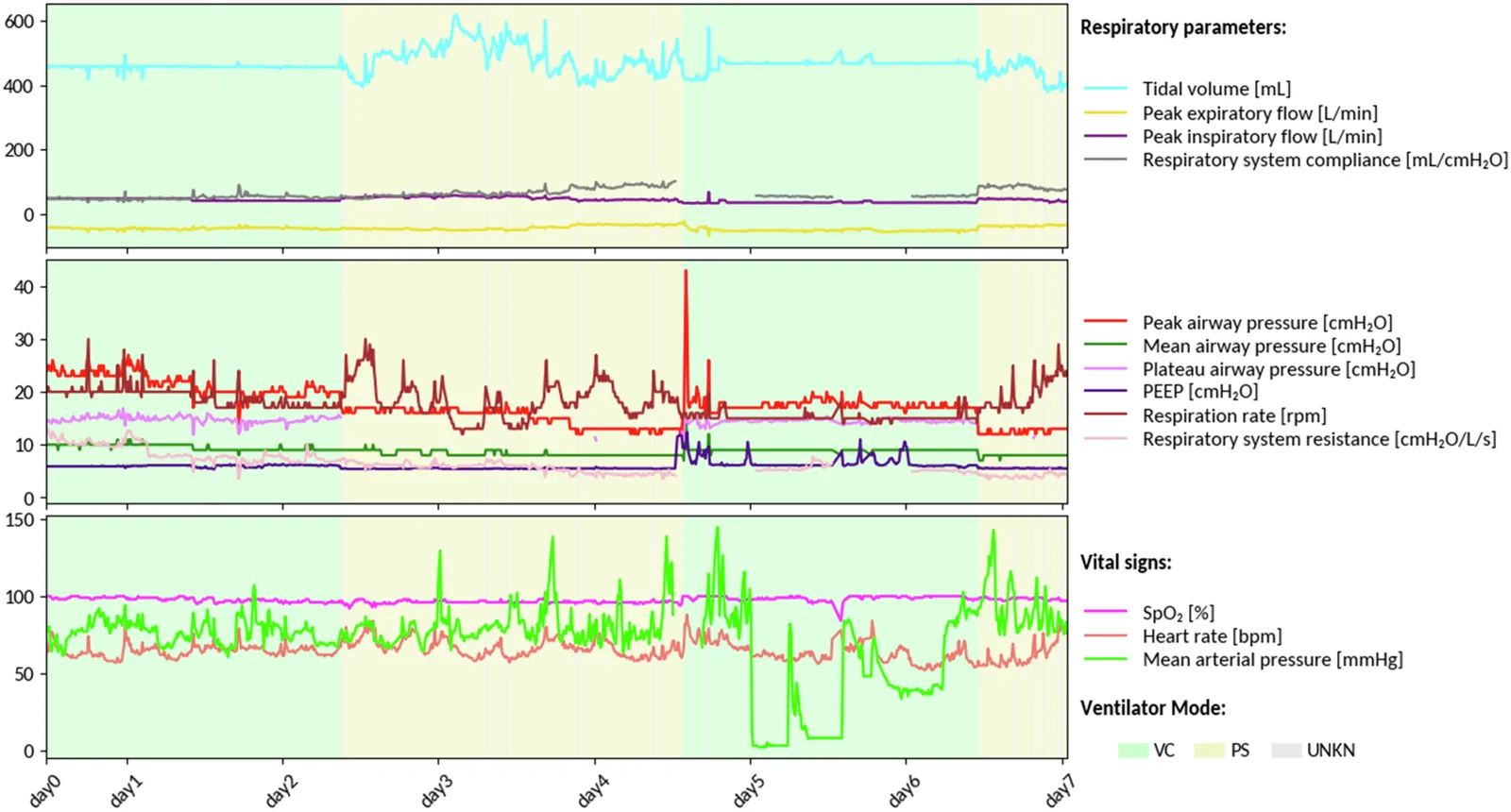
Comprehensive monitoring of respiratory parameters, pressure variables, and vital signs during MV. The figure displays three panels: (Top panel) Respiratory parameters including tidal volume (mL), peak expiratory flow (L/min), peak inspiratory flow (L/min), and respiratory system compliance (mL/cmH₂O). (Middle panel) Pressure variables including peak airway pressure (cmH₂O), mean airway pressure (cmH₂O), plateau airway pressure (cmH₂O), positive end-expiratory pressure maintained to prevent alveolar collapse (PEEP, cmH₂O), respiration rate (rpm) and respiratory system resistance (cmH₂O/L/s). (Bottom panel) Vital signs including peripheral oxygen saturation (SpO₂, %), heart rate (bpm) and mean arterial pressure (mmHg). Background colors indicate ventilator modes: VC (Volume Control) in green, PS (Pressure Support) in orange, and UNKN (Unknown) in gray.

**Fig. 5:**
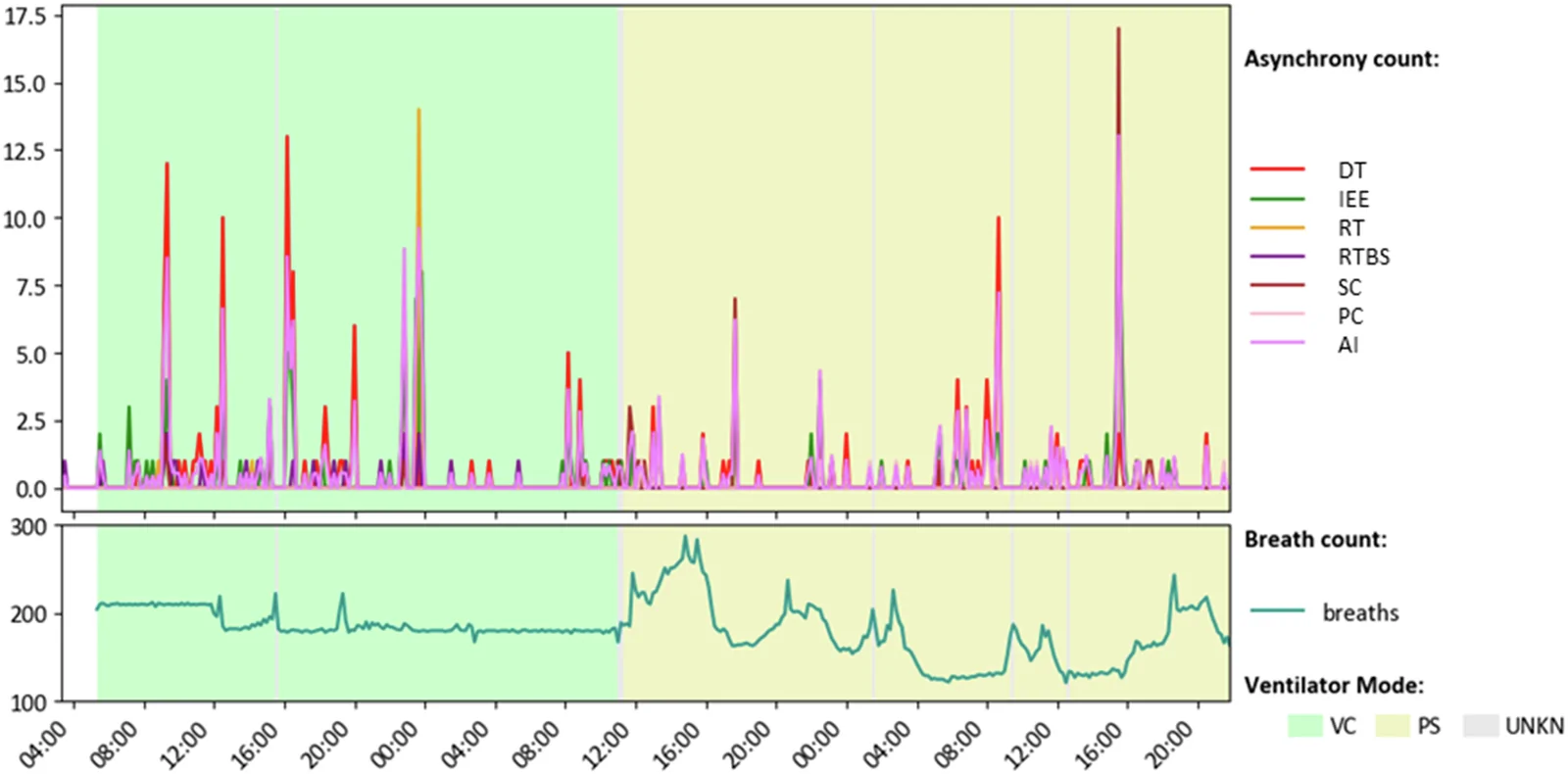
Representative case of asynchrony events during IMV. DT (Double Triggering), IE (Ineffective Effort), RT (Reverse Triggering), RTBS (Reverse Triggering with Breath Stacking), SC (Short Cycling), PC (Prolonged Cycling), and AI (Asynchrony Index). Total number of breaths during the analyzed window is shown in the bottom panel. Background colors indicate ventilator modes: VC (Volume Control) in green, PS (Pressure Support) in orange, and UNKN (Unknown) in gray.

Figure 4 shows a representative example of respiratory and vital signs variables continuously recorded during the 8-day ICU stay. Figure 5 shows a representative case with asynchrony event counts aggregated into 10-minute intervals over a 3-days period.

## Technical Validation

### Data quality assessment

A descriptive analysis was performed for each table to assess data quality and variable distributions. Statistical metrics including mean, median, mode, and the number of unique values per variable were calculated. Data completeness in waveform-derived tables should therefore be interpreted primarily as interval availability rather than missing values within pre-existing rows. Missing data were quantified by absolute counts and percentages of null values per column. These analyses were conducted using Python with the Pandas library.

### Data filtering

To ensure data quality and consistency, various filters were applied to the derived respiratory variables based on predefined thresholds validated by senior intensivist clinicians. Values falling outside these ranges, potentially due to sensor errors, artifacts, algorithmic inaccuracies, or acquisition issues, were excluded to minimize the presence of non-physiological data in the final dataset. The following thresholds were applied (variable names as shown in Fig. 1 [valid range]): peak airway pressure (*peak_press*): [0–80 cmH₂O], mean airway pressure (*mean_press*): [0–50 cmH₂O], stress index (*stress_index*): [0.5–1.5], peak inspiratory flow (*peak_flow*): [0–150 L/min], respiratory rate (*resp_rate*): [0–60 breaths/min], respiratory system resistance (*resistance*): [0–50 cmH₂O/L/s], respiratory system compliance (*compliance*) [0–150 mL/cmH₂O], tidal volume delivered per breath (*tidal_volume*): [0–2000 mL], hemoglobin oxygen saturation pulse oximeter (*spO₂*): [21–100%], mean arterial pressure (*mean_art_press*): [0–200 mmHg], peak expiratory flow (*peak_exp_flow*): [−150 to 0 L/min], heart rate (*heart_rate*): [0–300 bpm], plateau airway pressure (*plat_press*): [0–60 cmH₂O], and positive end-expiratory pressure (*peep*): [0–50 cmH₂O].

### Data completeness evaluation

**Fig. 6:**
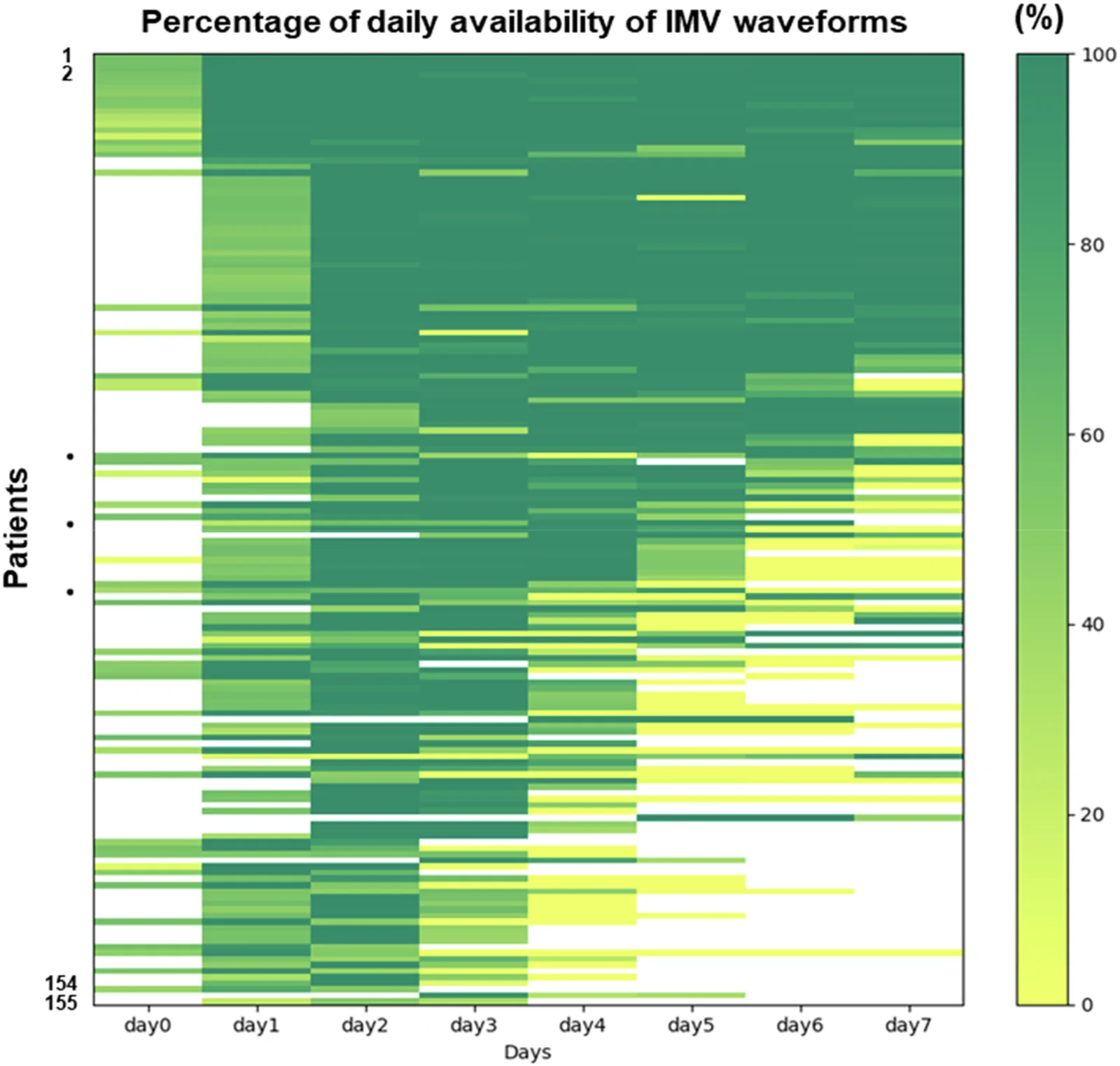
Heat map of daily availability of IMV waveform data during the first eight days following IMV initiation. Day0 corresponds with the day of patient intubation. The figure reflects day-level respiratory waveform coverage rather than completeness at the 10-minute interval level. RAMIC-I includes 13,433 hours of IMV recordings, representing 53.7% of the maximum achievable recording time across the cohort during the first 8-day after IMV initiation (25,032 hours, based on actual IMV duration capped at 8-day). This is lower than the absolute theoretical upper bound of 29,760 hours, assuming all 155 patients had remained ventilated for the full 8-day period. Reduced availability reflects both variability in IMV duration and periods of missing or unavailable waveform acquisition.

To visualize data completeness over time, Fig. 6 shows a heat map representing the availability of IMV waveforms per patient by day. Each row corresponds to a patient and each column to a day, with color intensity indicating the proportion of signals exhibiting variability. Dark green denotes 100% non-flat signals, light yellow indicates partial flat signals, and white represents days without data or with entirely flat signals. This visualization supports evaluation of data consistency and usability.

## Data Availability

Custom scripts used for preprocessing, physiological plausibility filtering and figure generation have been deposited in the public repository: https://dataverse.csuc.cat/file.xhtml?fileId = 462910&version = 2.0. These include the aggregation of breath-by-breath waveform data into non-overlapping 10-minute intervals, harmonization of missing-value representations, and deterministic filtering based on predefined physiological ranges. All preprocessing steps operate deterministically and do not involve manual or subjective transformations. Visualization scripts are provided for transparency regarding the quality-control and figure-generation workflow but do not modify the released dataset.
